# Improving Preclinic Preparation for Patients with Chronic Conditions in Quito, Ecuador: A Randomized Controlled Trial

**DOI:** 10.1155/2015/724245

**Published:** 2015-03-25

**Authors:** K. Rodriguez, E. Kaselitz, J. Wong, S. Ligard, D. Peck, V. Hugo Mena, F. Gordillo, D. Serlin, M. Heisler

**Affiliations:** ^1^University of Michigan Medical School, Ann Arbor, MI 48109, USA; ^2^Center for Clinical Management Research, Ann Arbor Veterans' Affairs (VA) Healthcare System, Ann Arbor, MI 48109, USA; ^3^Pontifical Catholic University of Ecuador (PUCE) Medical School, P.O. Box 17012184, Quito, Ecuador; ^4^Department of Health Behavior and Health Education, School of Public Health, University of Michigan, Ann Arbor, MI 48109, USA; ^5^Michigan Center for Diabetes Translational Research (MCDTR), University of Michigan, Ann Arbor VA, Ann Arbor, MI 48109, USA

## Abstract

*Objectives*. As in many settings, patients in community health centers in Ecuador do not complete previsit forms or receive assistance to identify questions and concerns they would like to address in brief clinic visits with physicians. We examined the comparative effectiveness of providing (1) a previsit form to complete; (2) a previsit form along with assistance in completing the form; and (3) usual care. *Methods*. Parallel, three-arm randomized controlled trial in two health centers serving indigent to low-income communities in Quito, Ecuador, among 199 adult patients who took medications for at least one chronic condition. Outcome measures were self-reported satisfaction with the visit, confidence in asking questions, and extent to which patients' objectives were met. *Results*. Patients who received assistance in completing a previsit form were more than twice as likely as participants in usual care to report achieving everything they wanted during their visit (AOR 2.2, *P* = 0.039). There were no differences in any outcomes between the groups who received the previsit form with no assistance and usual care. *Conclusions*. For high-quality patient-centered primary care, it is important to develop and test innovative and scalable interventions for patients and physicians to make the best use of limited clinic time.

## 1. Introduction

Since 2007, Ecuador has created a system of free, universal health care with government-funded primary care centers in low-income neighborhoods throughout the country. There is overwhelming demand for primary care services at these community health centers, and patients often experience long waits for their clinic visits. Moreover, because clinic visits are necessarily brief, clinicians often do not have sufficient time to address all patient concerns and needs. Such time constraints pose a significant challenge to providing patient-centered care [1].

In such contexts, it is critically important for patients to be well prepared for their primary care clinic visits, with a clear idea of the principal questions and concerns they would like to address. Research conducted in the United States using videotaped primary care encounters has shown that the first item addressed during the primary care visit receives the bulk of the attention (5 minutes versus 1.1 minutes for the next five out of six total topics addressed during the typical 15-minute visit) [2]. Other visit content analyses have found that the likelihood of preventive screening declines with each additional concern brought up by patients [3], and more complex patients are less likely to have medication dose changes [4]. The number and complexity of health issues and comorbidities a patient has—as well as any behavioral, emotional, and social issues they face—all added to the challenges of adequately addressing all of a patient's key concerns and needs [5, 6]. Low-income Ecuadorian adults with chronic illnesses often have low health literacy and face multiple social and other challenges to managing their conditions. Accordingly, effective ways to better enhance their ability to prepare to best convey to their provider their principal concerns for a primary care visit are especially needed [7].

Public primary health care centers in Ecuador, such as those serving the low-income communities of southern Quito, currently do not have any kind of previsit form for patients. Previsit forms can help patients identify their principal health concerns and questions, list their current prescribed medications and known medical conditions, and provide other basic information to help their providers prioritize patient concerns and topics to be addressed during office visits. In spite of long preclinic visit waits, patients currently do not have any preclinic visit preparation and do not complete previsit forms. Due to the high numbers of patients who often have long clinic waits for appointments at the local health centers, clinic waiting rooms are an ideal setting in which brief interventions are delivered to patients while they wait for care.

The basic concept of encouraging patients to prepare for their physician appointments is not new [8–12]. A number of studies have explored the impact of interventions and brief coaching on patient involvement and outcomes during clinic visits [12–15]. One pilot study found that sharing personalized, clinical information with patients before clinic visits improved doctor/patient communication and increased patient participation without increasing the length of visits [13]. However, a systematic review by Kinnersley and colleagues (2008) found that previsit interventions to promote question asking only provide a small benefit to patients, possibly because it is difficult to change established norms between patients and physicians during these encounters [14]. More research is needed on how to optimally help prepare patients, especially low-income patients with low health literacy who face multiple barriers to chronic disease management, for health care visits [9, 10, 16]. Truly patient-centered care requires that care interactions include consideration of patient priorities and preferences [17–19].

To address this need, we evaluated the effectiveness of a written previsit form for adults with chronic conditions to complete while waiting for their clinic visit to help prepare them to ask questions and provide information on their medical conditions and concerns to physicians. Because patients with little formal schooling may face literacy obstacles to completing a written form on their own, we also sought to assess outcomes from providing assistance to patients to complete the form. We hypothesized that patients who completed a brief written form on their own and patients who completed the form with the help of research staff before the clinic appointment would be more likely to report having their main health concerns and questions addressed in the subsequent clinic visit and thus improved satisfaction with the visit compared to patients receiving usual care. If we found improved outcomes, we could then make the form available to the clinic for continued use after the intervention.

## 2. Methods

### 2.1. Study Design and Setting

We conducted a parallel, randomized controlled trial (RCT) with scheduled patients waiting to see their primary care physicians in two community health centers serving indigent to low-income communities in southern Quito from June 2011 through August 2011. The patients were randomized to one of three arms: (1) usual care with no preclinic visit preparation; (2) provision of a brief written form for patients to complete on their own; or (3) provision of the written form with assistance from a member of the research staff in completing the form. The study received IRB approval from the University of Michigan, the Pontifical Catholic University in Quito, Ecuador, and the Ministry of Health in Ecuador. When adult patients arrived at the clinic for their appointment, they were told that the clinic was evaluating patients' experiences with clinic service and possible ways to improve service. They were then asked if they had any chronic conditions for which they had taken medications regularly for 3 months or longer to screen out patients who were recently diagnosed or were not getting regular care at the clinic. If they met this criterion, they were asked if they were willing to talk to a study team member to consider participating in an intervention study. If they agreed to participate, a study team member proceeded with oral informed consent and administered orally a brief baseline survey.

In order to avoid contamination, we randomized clinic sessions to one of the three study arms. Random sequence generation and treatment group assignment were determined centrally. The randomization was assigned weekly with each center carrying out one of the three study group conditions daily. Sequence was concealed until interventions were assigned. Data assessors were blinded to group assignment in analyses of the data. Depending on which of the three arms the clinic session had been randomized to that day, participants received no previsit form (usual care) (Group 1), a written previsit form to complete alone with brief oral instructions from a team member (Group 2), or a previsit form to be completed with the help of a team member (Group 3). In Group 3 the staff member reviewed the questions on the form orally with the patient and was available to help with form completion and questions while the participant was completing the form. This active assistance in completing the form took on average 5–10 minutes, and the most common questions concerned explanations of the questions on medication side effects, diet, and exercise. The research staff practiced their approach to providing oral assistance together before beginning the intervention to ensure uniformity across different staff. After their clinic visit, patients in all three groups were given the same instructions to complete a postclinic visit questionnaire administered orally by one of the study team. The study did not involve invasive procedures, medical recommendations, or review of medical records or other health record data.

### 2.2. Description of Previsit Form That Patients Completed to Prepare for Visit

The previsit form encouraged patients to think about what they wanted to achieve during the visit. It included a list of medical conditions for patients to check off for past medical history. It also included a list of signs and symptoms that patients could check off if they were experiencing any at the time of their visit. Technical terminology was avoided, and local colloquial terms were used (e.g.,* coto, vinagreras*) to facilitate patients' understanding. We also asked about their substance abuse history, as well as family history. There was a section for the patients to write in medical allergies and the list of their current medications, including prescription, over-the-counter, or any natural remedies. Below the list of medications, we asked if they were experiencing any side effects from the medication and if for any reason they had stopped taking one of their medications in the last 6 months. The form asked patients to write their chief concern they wanted to address in the visit and provided a space for them to list the questions and concerns they had for that day's clinic visit. To encourage patients to write down questions, examples were given such as “drug side effects.” Participants were also encouraged to show the form to their providers during the clinic visit (see Appendix A in the Supplementary Material available online at http://dx.doi.org/10.1155/2015/724245 for previsit form).

### 2.3. Study Measures

The baseline survey included age, gender, self-reported health status [20], education level, health literacy [21], comorbid conditions, chronic disease self-efficacy [22], level of patient activation [23], and understanding of and adherence to medications [24]. The survey also included patients' self-reported evaluations of their diet and exercise.

We examined three main study outcome measures from a survey orally administered immediately after participants' clinic visit. Individual items from the RAND Patient Satisfaction Questionnaire were examined [25]. These were as follows: (1) satisfaction with clinic visit “Are you completely satisfied with your clinic visit today?”; (2) whether the participants “achieved all that they had wanted to during the doctor's visit today”; and (3) whether the participants felt “confident about asking questions to the doctor” in the clinic visit (see Appendix B in the Supplementary Material for the baseline survey in Spanish and Appendix C in the Supplementary Material for the postvisit survey in Spanish). We also examined differences between Group 2 (previsit form only) and Group 3 (previsit form and assistance) in whether participants wrote down at least one question or concern that they wanted to discuss with their doctor during the upcoming clinic visit and mean number of questions or concerns listed on the form.

To determine differences in self-reported measures among the three groups, our recruitment goal was approximately 200 participants (approximately 65 participants per group). The xtmixed command in STATA, version 12.0 (StataCorp, College Station, TX), which fits multilevel mixed-effects linear regression models, was used to examine intervention effects.

## 3. Results

**Figure 1:**
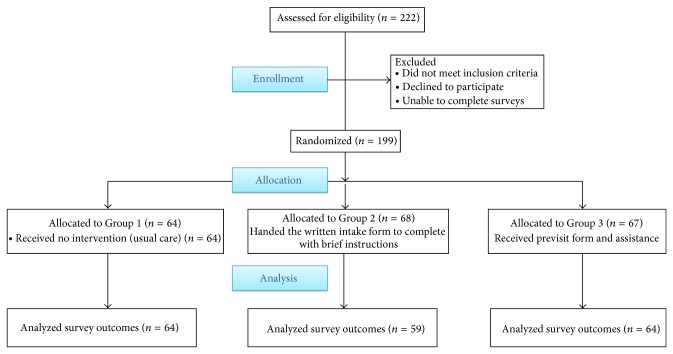
Flow of patients, Quito, Ecuador, 2011.

Figure 1 shows the flow of patients through the study. Of the 222 potential participants approached, 199 agreed to participate (90% participation). Consenting participants were randomly assigned to one of three groups: 64 were randomly assigned to the first group; 68 to the second group; and 67 to the third group. Of the 199 patients enrolled, 187 (94%) completed the post survey assessments.

Table 1 shows characteristics of participants who completed the initial survey (*N* = 199) and assessment results. The groups did not significantly differ in any measure, except for gender. Therefore, all analyses were adjusted for gender.

**Figure 2:**
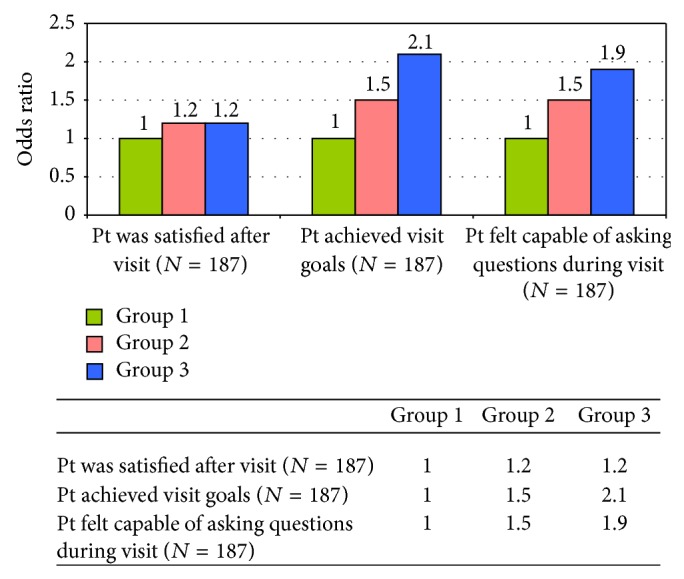
Intervention effects, Quito, Ecuador, 2011.

Figure 2 shows changes in study measures for participants between baseline and the postclinic visit measures and compares postclinic visit assessments among Group 1, Group 2, and Group 3. In analyses adjusting for gender, patients who received the revisit form along with assistance from a team member (Group 3) were more than twice as likely as participants in Group 1 to report that they had achieved everything they wanted during their visit (AOR 2.2, *P* = 0.039). While not statistically significant (*P* = 0.071) participants in Group 3 were 1.86 times as likely as Group 1 to report that they felt capable in their clinic visit of asking questions. Although a higher percentage of participants in Group 3 reported greater levels of satisfaction with their clinic visit than participants in Group 1, this difference also was not statistically significant. There were no differences between Groups 1 and 2 in any of our study outcome measures.

**Figure 3:**
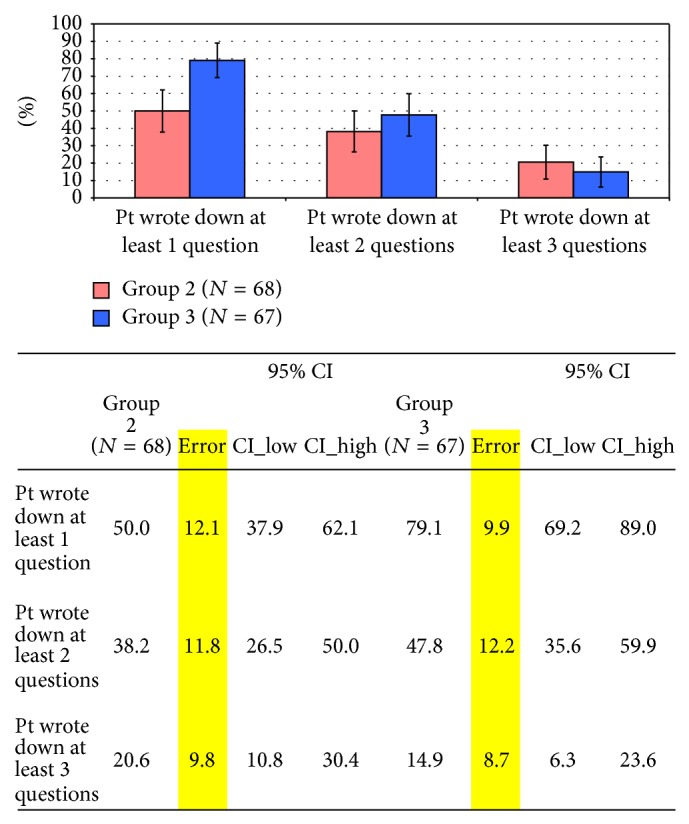
Intervention effect on writing down questions, Quito, Ecuador, 2011.

Figure 3 shows the comparison between the number of questions or concerns participants who received assistance in completing the form (Group 3) wrote down to discuss with their physician at the upcoming clinic visit compared to the number among participants who were just given the form to complete alone (Group 2). Patients who received help from a team member were more likely to write down at least one question (80% of patients in Group 3 wrote down at least one question or concern compared with 50% in Group 2, *P* < 0.01). Patients in Group 3 wrote down a mean of 1.6 questions compared to those in Group 2 who wrote down a mean of 1.1 questions.

## 4. Discussion

Among this sample of low-income Ecuadorian adult patients with very low levels of formal education and at least one chronic condition requiring medication at a government-funded community health center in southern Quito, patients who received assistance in completing a previsit form were more likely to engage in the consultation and to report accomplishing all their primary care visit goals and feeling capable of asking their physician questions at that visit than participants who did not receive any form or assistance. They were not, however, more likely to report feeling completely satisfied with their clinic visit than participants in the other groups. There were no differences in any measures between the group who received a previsit form but no assistance in completing the form and the group receiving no previsit forms or assistance.

Our findings reinforce evidence from observational and nonrandomized studies that suggest health benefits from receiving assistance in formulating questions and concerns for a clinic visit before the visit [9–12, 16]. Our findings also add to the literature by emphasizing ways such assistance may need to be adapted to meet the needs of low-literacy populations with low levels of formal education such as those served by many community health centers in low-income communities. Assistance in completing a previsit form appeared to be more effective than receipt of a written form alone in improving some postclinic visit outcomes, but not all outcomes. Because Group 3 combined assistance completing a previsit form with receipt of the form, we cannot determine whether orally eliciting patients' questions and concerns might have led to similar benefits without completion of the previsit form. Further research should explore reasons why providing the previsit form alone was not more effective than no previsit form and whether a different, simpler form could be effective. One possibility is that providing previsit forms alone is not effective among populations with high rates of low literacy. In our study sample, approximately 23% of participants in each group reported that they did not feel comfortable with filling out forms alone, and over 40% had only completed some primary school. Of note, however, these variables were not associated with whether patients wrote down at least one question or concern in Group 2, the group that received the previsit form alone. It is also worth noting that while most primary care practices in the United States routinely have patients complete revisit forms of some sort, there is little evidence from the United States or Europe on how completing previsit forms alone influences the subsequent primary care visit and discussion. There are, however, studies showing that providing cancer patients with question prompt sheets before oncology visits increases the number of questions patients ask in those visits [26–28].

Community health centers in southern Quito lack salaried staff who could provide assistance to patients in completing previsit forms. Thus, even though it only took 5–10 minutes to provide this assistance, different modalities to provide previsit coaching or assistance in formulating questions and concerns should also be explored. For example, as all the health centers in southern Quito have television screens and the capacity to show DVDs on these screens, it might be possible to develop an instructional DVD that could be shown in the waiting rooms to provide guidance to patients to think of questions and concerns they would like to discuss with their physician and to help in understanding how to fill out a previsit form.

Our study has a number of limitations. First, we only conducted a single preclinic brief intervention and only assessed participant-reported outcomes immediately after the clinic visit. Future studies should test previsit assistance interventions in this population over longer periods and include clinical outcomes along with patient-reported outcomes. Second, although we randomized clinic sessions to prevent contamination among the patients waiting to see the providers, the nature of the intervention prevented blinding of nurses and providers to the intervention itself. Moreover, it is possible that having patients in some clinic sessions bringing in the previsit form with listed questions and concerns may have influenced physician behaviors with patients who did not complete previsit forms. If anything, however, this would have introduced bias that would have reduced any differences between study groups. Third, this intervention focused exclusively on activating patients to discuss questions and concerns with their providers and did not address providers. Research by Chew-Graham and colleagues (2013) analyzing primary care visits for long-term health conditions found that patient concerns were often missed or disregarded by providers during clinic visits [29]. A more powerful intervention would also target provider behaviors to acknowledge and address patient concerns and questions they listed to be addressed at the clinic visit. The providers that participated in this intervention had a very favorable response to the previsit forms but were not given any guidelines on how they should use the forms during the visit, and no data was collected on how the forms influenced their behavior. Another significant limitation is that the form started with targeted questions about medical conditions, medications, and adherence before asking about more general questions and concerns the patient had for that clinic visit. This ordering and predominance of medical questions may have unintentionally restricted patients in the concerns they felt comfortable to bring up and failed to adequately encourage them to raise their own concerns that may have extended beyond a medical focus. Finally, we conducted this study among a predominantly female adult patient population in only two primary care clinics in one region of Quito. Our findings may not generalize to other populations and settings.

In conclusion, our study suggests that providing assistance with completing a previsit form designed to help patients formulate questions and concerns to discuss with their provider improved their confidence in asking questions and belief that they had achieved all their visit goals. Our study findings, however, do not support the provision of previsit forms alone for community centers serving low-income communities in which levels of formal education are low. Future research is required to determine what strategies would be necessary to improve benefits from providing a previsit form: providing more extensive initial orientation to completing such forms; making available staff or volunteers to provide ongoing assistance with completing the form such as what was provided in this intervention; or offering a simpler form that focused on eliciting the patient's main overall concerns and questions. A key question continues to be how best to both elicit patients' key priorities and concerns for primary care visits and also provide necessary information to primary care providers to meet the medical needs of patients with chronic conditions. To meet the goal of achieving high quality, patient-centered primary care in all settings throughout the Americas, it is critically important to continue developing and testing innovative and scalable interventions to better prepare both patients and their physicians to make the best use of the limited time in primary care visits [30, 31]. Our study findings support the hypothesis that providing active assistance to help patients identify their key questions and concerns before a clinic visit improves their visit satisfaction and belief that their objectives for the visit were met.

## Supplementary Material

The supplementary materials include the pre-visit form and surveys used in the study. Appendix A is the pre-visit form provided to patients to fill out before their visit with the provider. Appendix B is the baseline survey given to patients before the intervention, and Appendix C is the post-visit survey given to patients after the visit with their provider.

## Figures and Tables

**Table 1:** Patient population baseline characteristics (*N* = 199), Quito, Ecuador, 2011.

Characteristics	Group 1^a^ (*N* = 64)	Group 2^b^ (*N* = 68)	Group 3^c^ (*N* = 67)	Group 1 = 2 *P* value	Group 1 = 3 *P* value	Group 2 = 3 *P* value
General						
Age (mean)	58	59	57	0.65	0.72	0.41
Gender (% male)	9	28	16	0.01	0.23	0.11
Having a regular doctor (%)	56	60	55	0.69	0.33	0.17
Comfortable with filling out a form alone (%)	76	77	77	0.97	0.88	0.85
Schooling (%)				0.34	0.98	0.35
Some primary school (PK-7)	42	47	43			
Some secondary school (7–12)	41	44	37			
Completed secondary school (7–12)	11	6	16			
University (some/completed)	6	3	3			
Self-reported health status				0.51	0.91	0.43
Poor	25	34	27			
Fair	51	43	52			
Good	19	16	19			
Very good/excellent	5	7	4			
Baseline confidence to get questions answered by MD (%)				0.72	0.24	0.12
Not confident	11	12	10			
Fairly confident	30	32	22			
Confident	45	43	45			
Very confident	14	13	22			

^a^Usual care; ^b^group receiving form alone; ^c^group receiving form and assistance completing form.
