# Supplementary material for: Improving Preclinic Preparation for Patients with Chronic Conditions in Quito, Ecuador: A Randomized Controlled Trial
Source: Int J Family Med. 2015 Mar 25;2015:724245. doi: 10.1155/2015/724245 (PMC4390100; doi:10.1155/2015/724245)
Supplement: Supplementary file 1 — The supplementary materials include the pre-visit form and surveys used in the study. Appendix A is the pre-visit form provided to patients to fill out before their visit with the provider. Appendix B is the baseline survey given to patients before the intervention, and Appendix C is the post-visit survey given to patients after the visit with their provider. [file 724245.f1.pdf]

INTAKE FORM

ID: __________

1. *I have the following conditions:*

__ Anemia
__ Arthritis
__ Ashma/Rhinitis/Sinusitis
__ Cáncer
__ Depressión
__ Diabetes
__ Migraines
__ Anxiety
__ High Cholesterol
__ High Blood Pressure
__ Problems with your lungs
__ Problems with your heart
__ Problems with your kidneys
__ Liver disease
__ Thyroid problems
__ Gastritis
__ Obesity
__ Back Pain
__ Epilepsy
__ Other chronic conditions

2. *I have the following symptoms:*

__ Pain in _________
__ Sadness
__ Weakness
__ Diarrhea or constipation
__ Shortness of breath
__ Trouble breathing
__ Chest pain
__ Body aches
__ Difficulty urinating
__ Vaginal secretions
__ Vision problems
__ Tingling in the lower extremeties
__ Involuntary weight loss
__ Non-healing wounds
__ Acid reflux
__ Cough, greater than 2 weeks
Other: _________________

3. MEDICAL HISTORY

a. Occupation ____________________________
b. Do you smoke? Yes ☐1 No ☐2 How many cigarettes per day? __________
c. Do you drink alcoholic beverages? Yes ☐1 No ☐2 How often? ______________

d. Have you had any previous surgeries? List them.
____

e. Do you have any allergies to medication, food? List them.
____

4. Family History

Please write down any serious or chronic conditions in your family members.

a. Mother ____ I don't know ___
b. Father ____ I don't know ___
c. Children ____ I don't know ___
d. Siblings ____ I don't know ___
e. Maternal Granmother _____________________________________ I don't know ___
f. Maternal Grandfather ____________________________________ I don't know ___
g. Paternal Grandmother ____________________________________ I don't know ___
h. Paternal Grandfather _____________________________________ I don't know ___

5. MEDICINES (*OTC or prescription/herbal medicines/ alternative medicine*).

Please write down the names of the medication you have taken in the LAST WEEK.

a. Medication: (tablets, pills, injections, drops)
(1) ____ How many times a day?_________
(2) ____ How many times a day?_________
(3) ____ How many times a day?_________
(4) ____ How many times a day?_________
(5) ____ How many times a day?_________
b. Remedies:
(1) ____ How many times a day?_________
c. Treatments:
(1) ____ How many times a day?_________
d. I cannot recall the name:
(1) ____ How many times a day?_________

6. Are any of the medications you take bothersome in any way (pills, creams, injections, drops)?

Yes ☐1 No ☐2

7. If you answered yes, please write down the medicine and indicate how much it bothers you (not alot, some what, a lot) and in what way.

Medication	Not a lot	Some what	A lot	How does it bother you?
___________________	☐1	☐2	☐3	___________________
___________________	☐1	☐2	☐3	___________________
___________________	☐1	☐2	☐3	___________________
___________________	☐1	☐2	☐3	___________________

8. In the last 6 have you stopped taking your medication for any reason:

a. Unable to afford the medication Sí ☐1 No ☐2
b. Side effects Sí ☐1 No ☐2
c. Forgetfulness Sí ☐1 No ☐2
d. Fear of dependancy Sí ☐1 No ☐2
e. Fear that it can be harmful Sí ☐1 No ☐2
f. You felt better Sí ☐1 No ☐2
g. Different reason:_________________ Sí ☐1 No ☐2

9. What is the reason for your clinic visit today**?**
____
____
____
____

10. Please write down any questions/concerns you wish to discuss with your doctor:

a. **Regarding your medication:** (For example: reactions to your medications, how to take your medications, problems getting your medications)

____
____
____
____

b. **Other ways to improve your health:** (For example: increasing physical activity, improving your diet, decreasing alcohol intake, smoking cessation)

____
____
____
____

c. **Other important things: (**For example: emotional problems, any problems with your partner, family problems, laboratory test results)

____
____
____
____

11. If you wish, you may use these lines to write down anything else you want to bring up during your clinic visit today.

____
____
____
____
____
____
____
____
____

Pre-Visit Intake Form
ID ________________

1. What is your birth date? __________

2. What is your sex/gender? ☐1 Male ☐2 Female

3. Is this health center where you primarily get your care? ☐1 Yes ☐2 No

4. Do you have a personal physician assigned to you who provides most of your care? ☐1 Yes ☐2 No

5. When was the last time you came to this health center for a clinic visit for your own health care?

☐1 In the past three months
☐2 Between three and six months ago
☐3 Between six and 12 months ago
☐4 Over 12 months ago
☐5 This is my first clinic visit here

6. Do you know how to read and write? ☐1 Yes ☐2 No

7. What is the highest grade of school that you completed?

☐1 I did not complete primary education
☐2 I completed primary school
☐3 Less than HS graduate
☐4 High school graduate or GED
☐5 Some tech or vocational school
☐6 Some college or more

8. How confident are you filling out forms by yourself

☐1 Not at all
☐2 A little
☐3 Some what
☐4 Quite a bit
☐5 Extremely

9. In general, how would you rate your health?

☐1 Poor
☐2 Fair
☐3 Good
☐4 Very good
☐5 Excellent

10 For which health conditions do you or should you be taking medication?

____

____

Below is a list of problems that people sometimes have with their medicines. Please check how hard it is for you to do each of the following:

	Very Hard	Some what hard	Not Hard at All
11. Remember to take all the pills	☐1	☐2	☐3
12. Get your refills in time	☐1	☐2	☐3
13. Take so many pills at the same time	☐1	☐2	☐3

14. How well you know the instructions for taking your medication?

I do not know	A little	Some what	I know	I know very well
☐1	☐2	☐3	☐4	☐5

Please indicate how confident you are when aswering the following statements.

	Not at all confident	A little confident	Some what confident	Confident	Very confident
15. How confident are you in your ability to get a doctor to answer all your questions?	☐1	☐2	☐3	☐4	☐5
16. How confident are you in your ability to make the most of your visit with a doctor?	☐1	☐2	☐3	☐4	☐5
17 How confident are you in your ability to get a doctor to take your chief health concern seriously?	☐1	☐2	☐3	☐4	☐5

18. In the last 12 months, how often did your personal physician involve you in making decisions about your care as much as you wanted?

Never	Sometimes	Usually	Always	No visits in the last 12 months
☐1	☐2	☐3	☐4	☐5

19. In the last 12 months, how often did your personal physician seem to understand the kinds of problems you have in carrying out recommended treatments?

Never	Sometimes	Usually	Always	No visits in the last 12 months
☐1	☐2	☐3	☐4	☐5

Below are some statements that people sometimes make when they talk about their health. Please indicate how much you agree or disagree with each statement as it applies to you personally by checking your answer.

	Disagree Strongly	Disagree	Agree	Agree Strongly	N/A
20 When all is said and done, I am the person who is responsible for taking care of my health.	☐1	☐2	☐3	☐4	☐5
21. Taking an active role in my own health care is the most important thing that affects my health.	☐1	☐2	☐3	☐4	☐5
22. I am confident I can help prevent or reduce problems associated with my health.	☐1	☐2	☐3	☐4	☐5
23. I know what each of my prescribed medications do.	☐1	☐2	☐3	☐4	☐5
24. I am confident that I can tell whether I need to go to the doctor or whether I can take care of a health problem myself.	☐1	☐2	☐3	☐4	☐5
25. I am confident that I can tell a doctor concerns I have even when he or she does not ask.	☐1	☐2	☐3	☐4	☐5
26. I am confident that I can follow through on medical treatments I may need to do at home.	☐1	☐2	☐3	☐4	☐5
27. I understand my health problems and what causes them.	☐1	☐2	☐3	☐4	☐5
28. I know the different treatments that are available for my health problems.					

☐1 ☐2 ☐3 ☐4 ☐5

29. I am confident that I can maintain lifestyle changes, like eating right and exercising, even during times of stress.	Disagree Strongly	Disagree	Agree	Agree Strongly	N/A
	☐1	☐2	☐3	☐4	☐5

30. I am satisfied with my daily diet? ☐1 Yes ☐2 No

If you answered no, why not?____

31. I would like to improve my diet? ☐1 Yes ☐2 No

If you answered yes, state how you would like to improve (for example, I want to eat less fat, eat more fruit, drink less sodas)

____

32. I think I eat enough fruits? ☐1 Yes ☐2 No

33. Generally, how many servings of fruit do you in a day? (For example, one serving includes a medium-sized apple, a half-cup of grapes, or a quarter-cup of raisins): ______________________

34. I think I eat enough vegetables? ☐1 Yes ☐2 No

35. Generally, how many servings of vegetables do you in a day? (For example, a cup of carrots, a medium sized corn, a salad) ______________________________________

36. In the past 7 days, how many days did you eat meals high in fat, such as red meat or pork, whole milk, half and half, cheese, or fast food? ____ days (0–7)

37. In the past 7 days, how many days did you engage in physical activity (such as swimming, walking, running, riding a bike) in addition to work? ____ days (0–7)

38. In the past 7 days, how many days did you engage in at least 30 minutes of physical activity? Such as washing clothes, cleaning the house, walking to work). ____ days (0–7)

In the past 6 months, how difficult was it to follow through with your doctor's recommendations?

39. Exercising regularly:

- ☐1 Impossible
- ☐2 Very difficult
- ☐3 Difficult
- ☐4 Not very difficult

☐5 Easy
☐6 Does not apply to me

If you had problems following through, state why
____

40. Eating what the doctor recommended:

☐1 Impossible
☐2 Very difficult
☐3 Difficult
☐4 Not very difficult
☐5 Easy
☐6 Does not apply to me

If you had problems following through, state why
____

Please state if in the last 6 months:

41. The food you bought was not enough, and you did not have enough money to buy more.

☐1 Often
☐2 Some times
☐3 Never

42. You could not afford to buy meat, fish or eggs

☐1 Often
☐2 Some times
☐3 Never

Post-Visit Questionaire
ID: ____________

1. When thinking about your clinic visit today, indicate how much you agree or disagree with each statement:

	Disagree Strongly	Disagree	Neutral	Agree	Agree Strongly
a. I am completely satisfied with today's visit.	▢1	▢2	▢3	▢4	▢5
b. I accomplished everything I wanted to during today's visit.	▢1	▢2	▢3	▢4	▢5
c. The doctor provided enough time to explain the reaon for my clinic visit.	▢1	▢2	▢3	▢4	▢5
d. The doctor gave me all the information I needed regarding my health and my treatment.	▢1	▢2	▢3	▢4	▢5
e. The doctor explained what I need to do if my symptoms worsen.	▢1	▢2	▢3	▢4	▢5
f. I felt confident in asking my doctors questions.	▢1	▢2	▢3	▢4	▢5
g. My doctor took interest in all of the concerns I brought up today.	▢1	▢2	▢3	▢4	▢5

2. Is there something you wish the doctor would do differently next time? ▢1 Yes ▢2 No

____
____
____

3. From the questions you wrote down before your clinic visit:

QUESTION/CONCERN

____

____

a. Did you ask this question or discuss this issue with your doctor? ☐1 Yes ☐2 No
b. What is the treatment plan you and your doctor discussed regarding this issue?

   ____
c. Are you completely satisfied with this plan? ☐1 Yes ☐2 No
d. Do you still have questions regarding this issue? ☐1 Yes ☐2 No

   ____

QUESTION/CONCERN

____

____

a. Did you ask this question or discuss this issue with your doctor? ☐1 Yes ☐2 No
b. What is the treatment plan you and your doctor discussed regarding this issue?

   ____
c. Are you completely satisfied with this plan? ☐1 Yes ☐2 No
d. Do you still have questions regarding this issue? ☐1 Yes ☐2 No

   ____

QUESTION/CONCERN

____

____

a. Did you ask this question or discuss this issue with your doctor? ☐1 Yes ☐2 No
b. What is the treatment plan you and your doctor discussed regarding this issue?

   ____
c. Are you completely satisfied with this plan? ☐1 Yes ☐2 No
d. Do you still have questions regarding this issue? ☐1 Yes ☐2 No

   ____

4. Did you feel that filling out the questionnaire beforehand helped you get more out of your visit?

__ No, no help
__ Yes, helped a little
__ Yes, helped some what
__ Yes, helped a lot

5. Would you like to fill out a similar questionnaire every time you seek medical care to improve your clinic visits?

☐1 Yes ☐2 No

6. What changes would you recommend to improve the questionnaire you filled out? (For example, make the questionnaire shorter/longer, easier woring, use larger/smaller font, increase/reduce the help with filling out the questionnaire)

____

____
